# Bortezomib-mediated downregulation of S-phase kinase protein-2 (SKP2) causes apoptotic cell death in chronic myelogenous leukemia cells

**DOI:** 10.1186/s12967-016-0823-y

**Published:** 2016-03-09

**Authors:** Ahmad Iskandarani, Ajaz A. Bhat, Kodappully S. Siveen, Kirti S. Prabhu, Shilpa Kuttikrishnan, Muzammil A. Khan, Roopesh Krishnankutty, Michal Kulinski, Rihab R. Nasr, Ramzi M. Mohammad, Shahab Uddin

**Affiliations:** Translational Research Institute, Academic Health System, Hamad Medical Corporation, PO Box 3050, Doha, State of Qatar; Department of Anatomy, Cell Biology and Physiological Sciences, American University of Beirut, Beirut, Lebanon

**Keywords:** Proteasome pathway, Velcade^®^, SKP2, p27Kip1, Apoptosis

## Abstract

**Background:**

Proteasome inhibitors are attractive cancer therapeutic agents because they can regulate apoptosis-related proteins. Bortezomib also known as Velcade^®^, a proteasome inhibitor that has been approved by the food and drug administration for treatment of patients with multiple myeloma, and many clinical trials are ongoing to examine to the efficacy of bortezomib for the treatment of other malignancies. Bortezomib has been shown to induce apoptosis and inhibit cell growth of many cancer cells. In current study, we determine whether bortezomib induces cell death/apoptosis in CML.

**Methods:**

Cell viability was measured using MTT assays. Apoptosis was measured by annexin V/PI dual staining and DNA fragmentation assays. Immunoblotting was performed to examine the expression of proteins. Colony assays were performed using methylcellulose.

**Results:**

Treatment of CML cells with bortezomib results in downregulation of S-phase kinase protein 2 (SKP2) and concomitant stabilization of the expression of p27Kip1. Furthermore, knockdown of SKP2 with small interference RNA specific for SKP2 caused accumulation of p27Kip1. CML cells exposed to bortezomib leads to conformational changes in Bax protein, resulting in loss of mitochondrial membrane potential and leakage of cytochrome c to the cytosol. In the cytosol, cytochrome c causes sequential activation of caspase-9, caspase-3, PARP cleavage and apoptosis. Pretreatment of CML cells with a universal inhibitor of caspases, z-VAD-fmk, prevents bortezomib-mediated apoptosis. Our data also demonstrated that bortezomib treatment of CML downregulates the expression of inhibitor of apoptosis proteins. Finally, inhibition of proteasome pathways by bortezomib suppresses colony formation ability of CML cells.

**Conclusions:**

Altogether, these findings suggest that bortezomib suppresses the cell proliferation via induction of apoptosis in CML cells by downregulation of SKP2 with concomitant accumulation of p27Kip1, suggesting that proteasomal pathway may form novel therapeutic targets for better management of CML.

**Electronic supplementary material:**

The online version of this article (doi:10.1186/s12967-016-0823-y) contains supplementary material, which is available to authorized users.

## Background

Ubiquitin proteasome system (UPS), a multi-catalytic protein complex, plays critical roles in the regulation of key signaling proteins involved in many cellular functions [1–4]. Dysregulation of ubiquitin-dependent pathways has been implicated in the pathogenesis of many cancers [5–7]. SKP2, an SCF ubiquitin ligase belonging to the family of F-box proteins has been shown to be involved in cell cycle progression and cell proliferation [8–10]. It has been shown that SKP2 is overexpressed in many cancers [11–14]. SKP2 plays a rate-limiting factor in SCF protein complex that degrades p27Kip1 via ubiquitination and subsequently proteasome-mediated degradation [8–10]. Overexpression of SKP2 has been linked with poor prognosis of many cancers [15–18]. The significance of SKP2 in regulating p27Kip1 expression has been shown in various human malignancies, including diffuse large cell lymphoma and many solid tumors [19–23]. SKP2 gene silencing using small inference RNA (siRNA) in many cancer cell lines has led to the accumulation of p27Kip1, p21waf1 expression and inhibited cell growth and survival via cell cycle arrest and induction of apoptosis [8–10]. The anticancer agents induce cytotoxic effects mainly by inducing apoptotic cell death in various cancer cells. Growing number of studies have shown that proteasome inhibitors suppress the growth of many human cancer cells via induction of apoptosis mediated cell cycle arrest/cell death [24–27]. The bortezomib, a proteasome inhibitor has been approved for the treatment of multiple myeloma and mantle cell lymphoma in the United States by the US. Food and drug administration and for those patients who had already gone through one prior chemotherapeutic treatment. Several clinical studies are currently ongoing to determine the efficiency of bortezomib for the better management of other types of human cancers [28].

In the present study, our findings indicate that bortezomib-mediated inhibition of cell proliferation of chronic myelogenous leukemia cells (CML) cell lines is associated with down-regulation of SKP2 with concomitant up-regulation/stabilization of p27Kip1. Furthermore, CML cells treated with this particular proteasome inhibitor; bortezomib showed inhibition of cell growth, and induction of apoptosis by activation of the caspase cascade and disruption of the mitochondrial equilibrium. Altogether our findings suggest that bortezomib mediated downregulation of SKP2 is a novel effect in CML cells and implicates that proteasome inhibitors may have a novel therapeutic potential for the intervention of CML.

## Methods

### Reagents and antibodies

Bortezomib (Velcade^®^) and antibodies against Bax, tubulin, cytochrome c, GAPDH, caspase-3, caspase-9, and PARP were purchased from Santa Cruz Biotechnology, Inc. (Santa Cruz, CA, USA). Antibodies against BID and cleaved caspase-3 were purchased from Cell Signaling Technologies (Beverly, MA, USA). XIAP, cIAP1, and caspase-8 antibodies were purchased from R and D (USA). BD Cytofix/Cytoperm plus fixation and permeabilization solution kit with BD GolgiPlug, propidium iodide (PI) staining solution, annexin V binding buffer, mitochondrial membrane potential detection (JC-1) kit, stain buffer (FBS), annexin V-FITC antibody, H2AX (pS139)-Alexa Fluor 647 antibody, rabbit anti-active caspase-3- Bv605 antibody and PARP cleaved form-AF700 antibody were obtained from BD Biosciences (NJ, USA). Apoptotic DNA-ladder kit was obtained from Roche (Penzberg, Germany).

### Cell culture

K562, AR230, and LAMA-84 CML cells were grown in RPMI 1640 medium supplemented with 10 % (v/v) fetal bovine serum, 100 U/ml penicillin and 100 U/ml streptomycin at 37 °C in humidified atmosphere containing 5 % CO_2_.

### 3-(4, 5-Dimethylthiazol-2-yl)-2, 5-diphenyltetrazolium bromide assays

1 × 10^4^ cells were treated with the indicated doses of bortezomib in a final volume of 0.1 ml for 24 h. The ability of bortezomib to suppress cell growth was determined by MTT cell proliferation assays, as previously described [29]. Replicates of three wells for each dosage including vehicle control were analyzed for each experiment.

### Cell cycle analysis

To determine whether bortezomib can induce cell cycle arrest in CML cells, K562, and AR230 cells were exposed to various doses of bortezomib for 24 h. Cells were washed, fixed with 70 % ethanol, and incubated for 30 min at 37 °C with 0.1 % RNase A in phosphate-buffer saline (PBS). Cells were washed again, resuspended, and stained with 25 μg/ml propidium iodide (PI) for 30 in PBS min at room temperature. Cell distribution across the cell cycle was analyzed by flow cytometry using BD LSRFortessa analyzer (BD Biosciences) as described previously [30].

### Annexin V/propidium iodide dual staining

K562 and AR230 cells were incubated with the indicated concentrations of bortezomib. The cells were collected by centrifugation, washed with PBS and stained with fluorescein-conjugated annexin V and propidium iodide (BD Biosciences) and the percentage of cells undergoing apoptosis was measured by flow cytometry as previously described [31].

### Knock down study using SKP2 siRNA

SKP2 siRNA and scrambled control siRNA were obtained from Qiagen. K562 and AR230 cells were transfected using Lipofectamine 2000 reagent (Invitrogen) according to the manufacturer’s instructions. Six hours post-transfection, the lipid and siRNA complex were removed, and fresh RPMI medium with serum was added. After 48 h of incubation, cells were lysed and immunoblotted with antibodies against SKP2, p27Kip1, and GAPDH as a loading control.

### Cell lysis and immunoblotting

CML cells were treated with bortezomib for various time periods as described in the figure legends and lysed as described previously [32]. Proteins (25–50 µg) were resolved by SDS-PAGE and transferred to polyvinylidene difluoride (PVDF) membrane (Immobilon, Millipore, Billerica, MA). Immunoblotting was performed using various antibodies, the blots were developed and further visualized under a ChemiDoc system (Amersham, Bio-Rad, USA).

### Quantitation of DNA double strand breaks (H2AX)

K562 cells were treated with various doses of bortezomib for 24 h. After the incubation period, the cells were fixed and permeabilized using BD Cytofix/Cytoperm plus fixation and permeabilization solution kit, as per protocol from the manufacturer. Approximately 0.3 × 10^6^ cells in FBS were stained with 5.0 µl of H2AX (pS139)-Alexa Fluor 647 antibody for 30 min at room temperature. The cells were washed once with FBS (300 g for 5 min) and then resuspended in FBS. The DNA breaks were measured by flow cytometry using BD LSRFortessa analyzer.

### DAPI staining

10^5^ cells of K562 CML cell line were incubated in the presence of different concentrations of bortezomib (10, 25 and 50 nm) in a final volume of 1 ml per condition for 24 h. The cells were then counted using hemacytometer in duplicates and 6 × 10^4^ cells were washed with 1 × PBS, centrifuged at 1200 rpm for 5 min. The pellet then was resuspended in 150ul of 1× PBS and using the Cytofunnels set we did cytospining of the samples on the microscopic slides at 800 rpm for 5 min. Furthermore, the cells were fixed with 100 % methanol for 30 min at −20 °C followed by permeabilization for 5 min at 4 °C in 0.2 % Triton X-100 solution in PBS. Cells were then washed and nuclei were counterstained with 1 µg/ml DAPI in PBST for 5 min and rinsed 3 times in PBST and once in H_2_O to remove salts. Slides were then mounted using Vectashield antifade mounting medium (Vector Laboratories, CA, USA) and covered with cover glasses. Excess of fluid was removed with paper towel and slides were sealed with transparent histofluid and kept overnight at 4 °C. Imaging was performed the next day on Zeiss axio imager microscope using 63×/1.25 oil objectives.

### Mitochondrial membrane potential measurement

K562 cells were treated with bortezomib for 24 h, briefly stained with JC-1 stain for 15 min in dark at 37 °C as per instructions from the kit manufacturer. The cells were washed with 1× assay buffer, re-suspended using 500 µl of the same buffer. After that mitochondrial membrane potential was measured by flow cytometry using a BD LSRFortessa analyzer (BD Biosciences, USA) [33].

### Flow cytometric analysis of active caspase-3 and cleaved PARP

K562 cells were treated with various doses of bortezomib for 24 h, cells were fixed and permeabilized using BD Cytofix/Cytoperm plus fixation and permeabilization solution kit, as per protocol from the manufacturer. Approximately 0.3 × 10^6^ cells in FBS were stained with 5.0 µl each of anti- Active Caspase-3-BV605 and PARP Cleaved Form-AF700 antibodies for 30 min at room temperature. Cells were then washed with FBS and then resuspended using the same buffer. Quantification of the active caspase-3 and cleaved PARP in the cells were analyzed by flow cytometry using BD LSRFortessa analyzer.

### Measurement of cytochrome c release

To measure the release of cytochrome *c* from mitochondria, we performed the assay as reported earlier [34]. K562 cells were treated with 10, 25 and 50 nm bortezomib for 24 h, cells were harvested and resuspended in hypotonic buffer (1 mM Tris–HCl, pH 7.4, 0.13 M NaCl, 5 mM KCl, 7.5 mM MgCl_2_). Cells were homogenized and centrifuged to obtain the cytosolic as well as mitochondrial fractions. Twenty to twenty-five microgram of protein from cytosolic and mitochondrial fractions of each sample were analyzed by immunoblotting using an anti-cytochrome c and tubulin antibody.

### Clonogenic leukemic assays using methylcellulose

K562, AR230 and LAMA84 (1 × 10^4^) cells were treated with and without bortezomib as described in the figure legends and mixed with 1.0 mL of MethoCult H4034 Optimum (Stem Cell Technologies). Colonies were counted based on morphology after 10 days.

### Statistical analysis

Comparisons between groups were made using the paired Student’s *t* test. The software GraphPad Prism (version 5.0 for Windows, GraphPad Software Inc., San Diego, CA, http://www.graphpad.com). Values of * p < 0.05 were considered statistically significant.

## Results

### Bortezomib is antiproliferative and induces apoptosis in CML cells

**Fig. 1:**
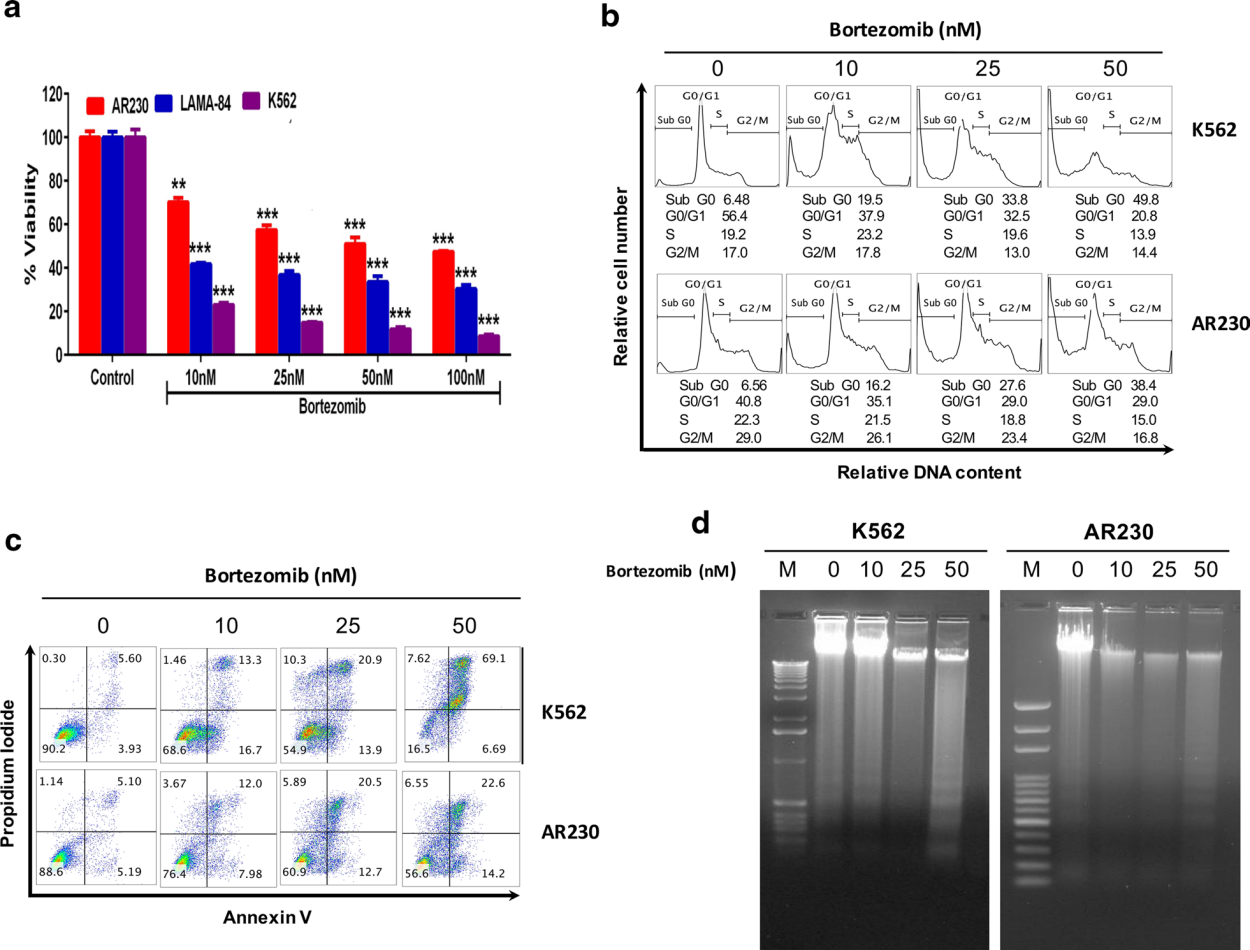
Effects of Bortezomib on proliferation, cell cycle progression, and apoptosis in CML cells. **a** Bortezomib inhibits the cell viability of CML cells. AR230, LAMA-84 and K562 cells were incubated with 10, 25, 50 and 100 nm bortezomib for 24 h. Cell proliferation assays were performed using MTT as described in “Methods” section. The* graph* displays the mean ± SD (standard deviation) of three independent experiments with replicates of six wells for all the doses. **p < 0.01, ***p < 0.001 **b** Bortezomib induces the increase of subG0 population of CML cells. K562 and AR230 cells were treated with 10, 25 and 50 nm of bortezomib for 24 h. Thereafter, the cells were washed, fixed and stained with propidium iodide, and analyzed for DNA content by flow cytometry as described in “Methods” section. **c** Bortezomib induces apoptosis in CML cells. K562 and AR230 cells were treated with 10, 25 and 50 nm of bortezomib for 24 h and cells were subsequently stained with flourescein-conjugated annexin-V and propidium iodide (PI) and analyzed by flow cytometry. **d** Bortezomib treatment of CML cells induces DNA fragmentation. K562 and AR230 cells were treated with 10, 25 and 50 nm bortezomib as indicated for 24 h and DNA was extracted and separated by electrophoresis on 1.5 % agarose gel

To assess the effect of bortezomib on cell viability, a panel of human CML cell lines (AR230, LAMA-84, and K562) were treated with increasing concentrations (10, 25 and 50 nm) of bortezomib for 24 h. A dose-dependent decrease in cell proliferation was observed in all the treated cell lines (Fig. 1a). Bortezomib-mediated inhibition of cell viability was also observed in a time-dependent manner (data not shown).

To investigate whether the inhibition of cell viability induced by bortezomib is due to cell cycle arrest or apoptosis K562 and AR230 cells were treated with different doses of bortezomib for 24 h as indicated. An increase in subG0 population was observed in a dose-dependent manner with the cell lines, K562, and AR230 (Fig. 1b). The sub-G0 population of cells was found to increase from 6.48 % in control cells to 19.5, 33.8 and 49.8 % at 10, 25 and 50 nm bortezomib-treated K562 cells respectively. Similar results were obtained in AR230 cells with an increase of sub-G0 population from 6.56 % in control cells to 16.2, 27.6 and 38.4 % in cells treated with 10, 25 and 50 nm of bortezomib respectively. The increase in sub-G0 population was accompanied by decreased G0/G1 and G2/M phases in bortezomib-treated CML cells.

To investigate whether the increased sub-G0 population in response to bortezomib treatment in CML cells was a resultant of induction of apoptosis, K562, and AR230 cells were treated with 10, 25 and 50 nm bortezomib for 24 h and apoptosis was measured by annexin-V-FITC/PI dual staining. As shown in Fig. 1c, Additional file 1: Figure S1a, b, treatment of CML cells with bortezomib resulted in a dose-dependent increase in apoptosis. Furthermore bortezomib treatment resulted in an increase in early (annexin +ive and PI −ive cells) and late (annexin +ive and PI +ive cells) stage apoptotic cell fractions in K562 and AR230 respectively. There was significant bortezomib-mediated apoptosis in K562 cell (18 % at 10 nm, p < 0.05; 40.1 % at 25 nm, p < 0.001, 81.1 % at 50 nm, p < 0.001) and in AR230 cells (13.5 % at 10 nm, p < 0.001; 33.2 % at 25 nm, p < 0.001, 38.4 % at 50 nm, p < 0.001) with respect to the untreated control cells. Bortezomib treatment was found to be more effective towards K562 cells line when compared to AR230 cells with all tested doses (Additional file 1: Figure S1c, d). To further confirm the bortezomib-mediated apoptosis in K562 and AR230 cells, we performed the DNA fragmentation analysis as described in the “Methods” section. An increase in DNA fragmentation was observed upon bortezomib treatment of both cell lines in a dose-dependent manner (Fig. 1d). These results correlated with the quantification of phospho-γ-H2AX (S139) by flow cytometry as shown in Additional file 2: Figure S2a. Bortezomib treatment causes a significant increase in DNA double strand breaks as evident by increase in phosphorylation of H2AX. DNA damage leads to double-stranded breaks that are always followed by the phosphorylation of the histone H2A variant, H2AX. Since phosphorylation of H2AX at Serine 139 is abundant, fast, and directly correlates well with each double-stranded break, it is used as a sensitive marker for DNA damage [35]. Furthermore, we performed DAPI immunofluorescence staining to visualize apoptotic nuclear bodies—a common hallmark of apoptosis. As shown in Additional file 2: Figure 2b, bortezomib treatment of K562 cells causes a dose-dependent malformation of nuclei architecture followed by subsequent formation of apoptotic bodies. The results suggest that the antiproliferative activity of bortezomib in CML cells is via induction of apoptosis.

### Bortezomib inhibits proteasome activity via SKP2 downregulation in CML cells

**Fig. 2:**
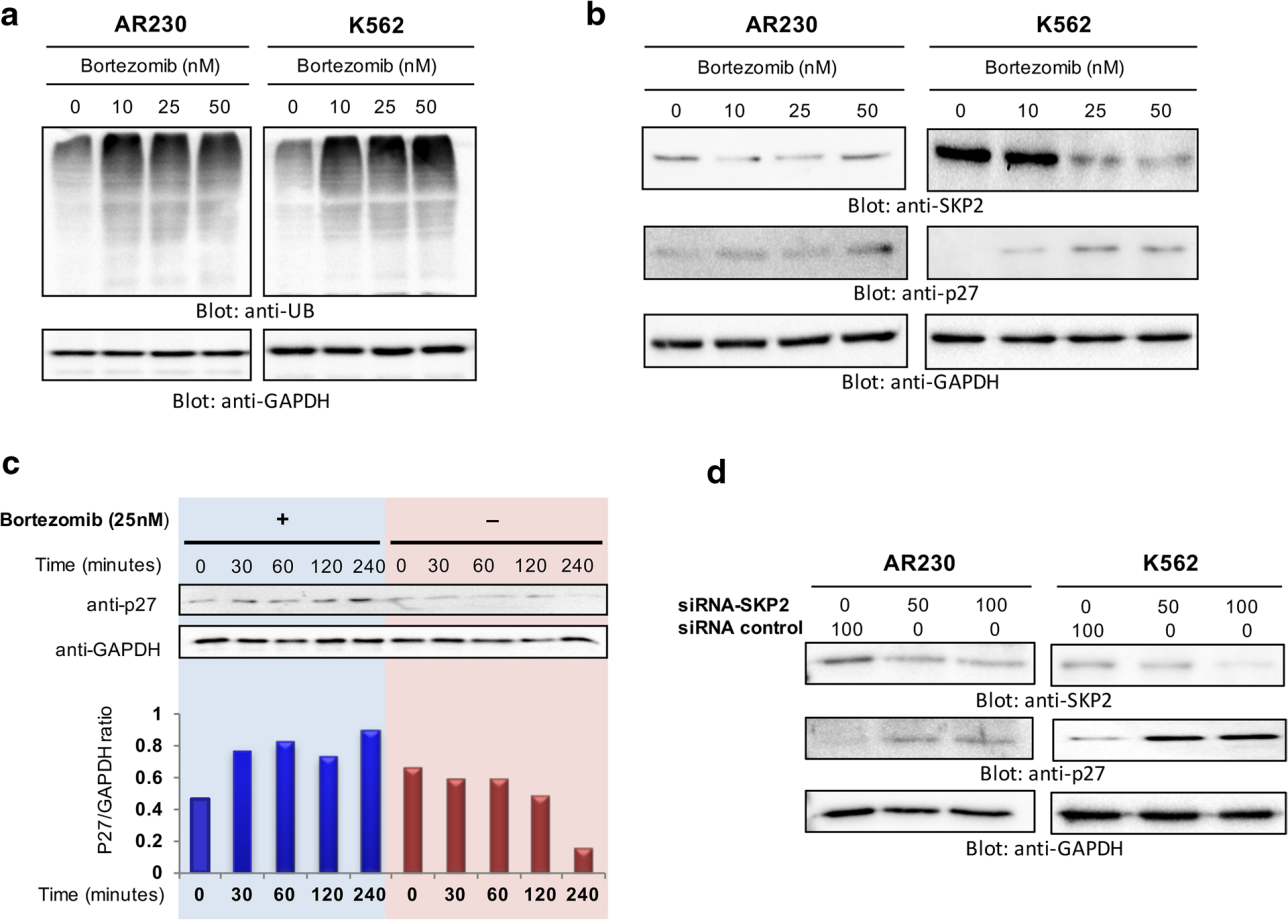
Downregulation of SKP2 pathway by proteasome inhibition causes accumulation of ubiquitinated proteins and upregulates the expression of p27Kip1. **a** Bortezomib-mediated ubiquitination of various proteins in CML cells. AR230 and K562 cells were treated with different doses of bortezomib for 24 h as indicated. After cell lysis, equal amounts of proteins were separated by SDS–PAGE, transferred to PVDF membrane, and immunoblotted with antibodies of anti-ubiquitin and GAPDH as indicated **b** Bortezomib treatment down-regulated the expression of SKP2 and increased the level of p27Kip1. AR230 and K562 cells were treated with various doses of bortezomib for 24 h as indicated. After cell lysis, equal amounts of proteins were separated by SDS-PAGE, transferred to PVDF membrane, and immuno-blotted with antibodies against SKP2, p27Kip1 and GAPDH as indicated. **c** Bortezomib treatment of K562 cells causes the stabilization of p27. K562 cells were treated with and without 25 nm of Bortezomib for 24 h. Cells were then treated with 10 μM Cyclohexamide for 30, 60, 120 and 240 min. Cells were lysed and equal amounts of proteins were separated by SDS-PAGE, transferred to PVDF membrane, and immuno-blotted with antibodies against p27Kip1 and GAPDH as indicated. Each band was quantified by densitometry and ratio of p27/GAPDH was plotted. **d** SKP2 siRNA expression downregulates SKP2 and accumulated p27Kip1. AR230 and K562 cells were transfected with Scrambled siRNA (100 nm) and SKP2 siRNA (50–100 nm) using Lipofectamine 2000 as described in “Methods” section. After 48 h of transfection, cells were lysed and equal amounts of proteins were separated by SDS-PAGE, transferred to PVDF membrane, and immunoblotted with antibodies against SKP2, P27Kip1, and GAPDH as indicated

During growth and division, the cell requires a periodic activation of a family of protein kinases known as several cyclin-dependent kinases (CDK), whose expression levels are highly regulated by ubiquitination and proteasomal degradation [36–39]. Several studies have reported that the expression of p27Kip1, a CDK inhibitor, is dysregulated by proteasome pathway [40–42]. The F-box protein, SKP2 is an ubiquitin ligase that plays a vital role in the degradation of p27Kip1 [15, 19, 20]. In accordance with these facts, we intend to check whether bortezomib-mediated apoptosis is due to proteasome inhibition and degradation of SKP2. The proteasome inhibition in K562 and AR230 cells mediated by bortezomib treatment after 24 h was evidenced by the accumulation of polyubiquitinated proteins (Fig. 2a).

Moreover, it was observed that bortezomib treatment resulted in downregulation of SKP2 in both cell lines, with a concomitant up-regulation of p27Kip1 (Fig. 2b). Similar results were obtained using another CML cell line, LAMA-84 (Additional file 3: Figure S3). This antagonistic effect observed for SKP2 and p27Kip1 suggests that bortezomib induced- downregulation of SKP2 forms an important mechanism to induce apoptosis in CML cell lines via up-regulation of p27Kip1. Since the p27Kip1 abundance increased as a result of bortezomib treatment of CML cells, we investigated the effect of bortezomib on the stability of p27Kip1 using cycloheximide chase assay. As indicated in Fig. 2c, bortezomib treatment increased the stability of p27Kip1 in K562 cells. These findings suggest that bortezomib-mediated p27Kip1 up-regulation is a result of p27Kip1 stabilization.

To further confirm the antagonistic effect observed for SKP2 and p27Kip1, we sought to silence the SKP2 expression in K562 cells by specific siRNA against SKP2. We observed that the knockdown of SKP2 resulted in the increased expression of p27Kip1 in CML cells (Fig. 2d). Altogether, the results suggest that the bortezomib-induced apoptosis is mediated by the downregulation of SKP2 and concomitant accumulation of p27Kip1 in CML cells.

### Bortezomib-induced apoptosis: involvement of mitochondrial pathway and activation of caspases

**Fig. 3:**
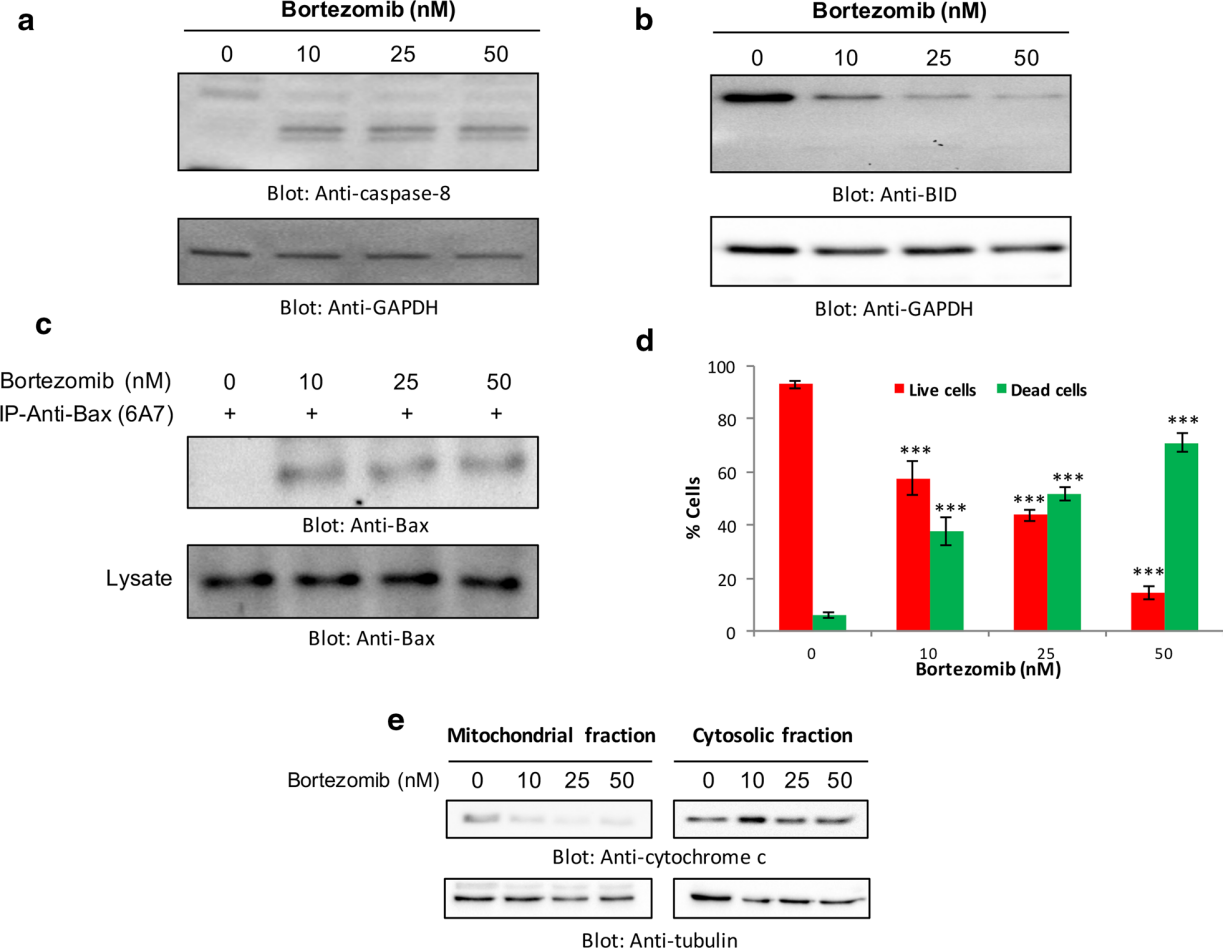
Bortezomib-induced activation of caspase-8 and mitochondrial apoptotic pathway. Bortezomib-induced activation of caspase-8 **a** and BID **b** in CML cells. K562 cells were treated with 10, 25, and 50 nm bortezomib for 24 h. Cells were lysed and 25 μg of protein were separated by SDS-PAGE, transferred to PVDF membrane, and immuno-blotted with antibodies against caspase-8, BID, and GAPDH. **c** Bortezomib-induced Bax conformation change in CML cells. After treating with 10, 25, and 50 nm bortezomib for 24 h, K562 cells were lysed in 1 % Chaps lysis buffer and subjected to immunoprecipitation with anti-Bax (6A7) antibody for detection of conformational changed Bax protein. In addition, the total cell lysates were applied directly to SDS-PAGE, transferred to PVDF membrane and immuno-blotted with anti-Bax polyclonal antibody (lower blot). **d** Bortezomib treatment causes the loss of mitochondrial membrane potential in CML cells. K562 cells were treated with 10, 25 and 50 nm of bortezomib for 24 h. Live cells with intact mitochondrial membrane potential (*red bar*) and dead cells with lost mitochondrial membrane potential (*green bar*) were measured by JC1 staining and analyzed by flow cytometry as described in “Methods” section. ***p < 0.001. **e** Bortezomib-induced release of cytochrome c. K562 cells were treated with 10, 25 and 50 nm of bortezomib for 24 h. Cytoplasmic and mitochondrial fractions were isolated as described in “Methods” section. Cell extracts were separated on SDS-PAGE, transferred to PVDF membrane, and immunoblotted with an antibody against cytochrome c and tubulin

Activation of caspase-8 followed by truncation of Bid leads its translocate to the mitochondrial membrane which results in activation of the intrinsic apoptotic pathway. In accordance to that, we sought to determine, whether bortezomib-induced apoptosis involves the mitochondrial-mediated activation of caspases. Our data suggest that K562 cells treated with bortezomib for 24 h showed activation of caspase-8 (Fig. 3a), followed by the truncation of Bid, which led to the conformational change of Bax protein as shown by the increase in activated form of Bax (6A7) in Fig. 3b, c.

**Fig. 4:**
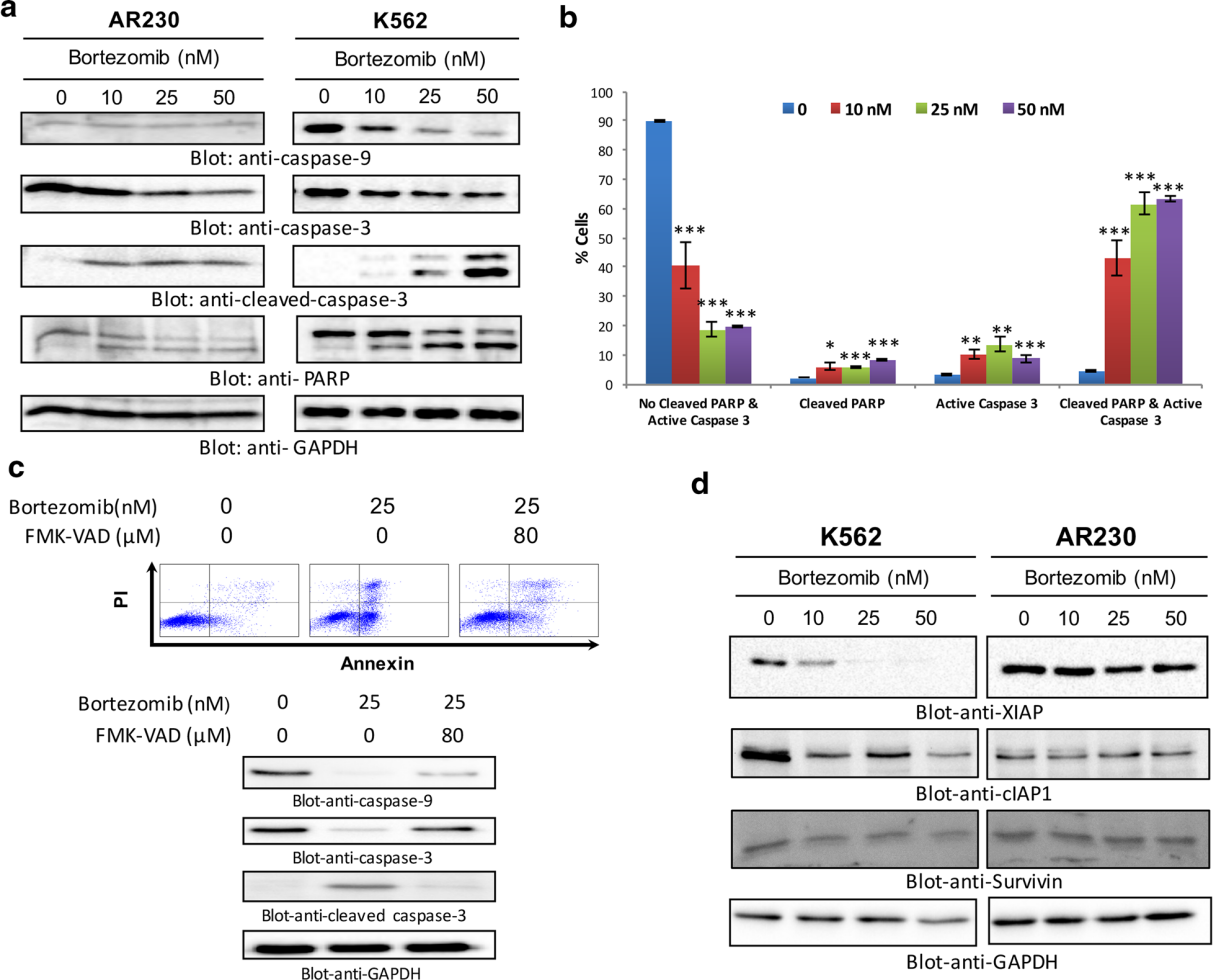
Activation of caspase-9, caspase-3, and cleavage of PARP induced by bortezomib treatment in CML cells. **a** Bortezomib mediated activation of caspase cascade in CML cells. AR230 and K562 cells were treated with and without 10, 25 and 50 nm of bortezomib for 24 h. Cells were lysed and 25 μg of proteins were separated on SDS-PAGE, transferred to PVDF membrane, and immunoblotted with antibodies against caspase-9, caspase-3, cleaved caspase-3, PARP and GAPDH. **b** Bortezomib-induced activation of caspase-3 and PARP cleavage in K562 cells was determined by flow cytometry as described in “Methods” section. *p < 0.05, ***p < 0.001. **c** Effect of z-VAD-fmk on bortezomib-induced apoptosis and activation of caspase-3. K562 cells were pre-treated with 80 μM z-VAD/fmk for 2 h and subsequently treated with 25 nm for 24 h. Cells were divided in two parts. In one part apoptotic cells were measured with fluorescein-conjugated annexin-V and propidium iodide (PI) and analyzed by flow cytometry (*upper panel*). The other part of the cells were lysed and 20 μg protein were separated by SDS-PAGE, transferred to PVDF membrane, and immunoblotted with antibodies against caspase-9, pro-caspase-3, cleaved caspase-3, and GAPDH (*lower panel*). **d** Bortezomib down-regulates expression of XIAP in CML cells. K562 and AR230 cells were treated with 10, 25 and 50 nm bortezomib for 24 h. Following incubation, cells were harvested and proteins were isolated separated on SDS-PAGE and immunoblotted with antibodies against XIAP, cIAP1, Survivin and GAPDH as indicated (representative of several experiments)

The altered conformation of Bax protein has been shown to reduce the mitochondrial membrane potential (MMP), leading to matrix remodeling resulting in cytochrome c release. We, therefore, investigated whether the bortezomib has an effect on the MMP in K562 cells. Cells were treated with increasing concentration of bortezomib and labeled using JC1 dye and MMP was measured by flow cytometry. JC-1 is a lipophilic, cationic dye that can specially enter into mitochondria. In control cells with elevated mitochondrial membrane potential, the dye forms J-aggregate complex with intense red fluorescence, while in apoptotic cells with reduced MMP, it gives a green fluorescence. Figure 3d illustrates a dose-dependent loss of MMPs (p < 0.001 in at all doses) in CML cells as indicated by the increase in JC1 green fluorescence—a marker of apoptotic cells. Similar results were obtained by using Rhodamine 6G, another dye utilized for the detection of mitochondrial membrane potential (data not shown). We then investigated the release of cytochrome c from the mitochondria in bortezomib-treated cells. As is evident in Fig. 3e cytochrome c was found to be released into the cytosol after bortezomib treatment. There was a concomitant decrease in cytochrome c level in the mitochondrial fraction of K562 cells. The results indicate that bortezomib-mediated inhibition of proteasome pathways led to the depolarization of MMP, which in turn led to the increased flux of cytochrome c in the cytosol. We further investigated whether the bortezomib-mediated release of cytochrome c can activate caspase-9, subsequently caspase-3 and eventually PARP cleavage. Figure 4a shows that bortezomib treatment of K562 and AR320 cells resulted in the activation of caspase-9, caspase-3, and cleavage of PARP. Similar results were obtained with LAMA-84 cells (Additional file 4: Figure S4). Furthermore, activation of caspase-3 and PARP cleavage was determined by flow cytometry. The data was in accordance with Western blot analysis showing significant caspase-3 activation and PARP cleavage in bortezomib-treated CML cells (Fig. 4b). In addition, pre-treatment of K562 cells with z-VAD-FMK, a generalized inhibitor of caspases, prevented bortezomib-mediated activation of caspase-3 and apoptosis (Fig. 4c), suggesting the involvement of caspases in bortezomib-mediated apoptosis.

Inhibitors of apoptosis proteins (IAP) have been shown to have direct effects on caspases [43]. Therefore, we investigated whether bortezomib-induced cell death involved modulation of IAP members which determines the cell’s response to the apoptotic signal. Therefore, we sought to determine whether bortezomib has any effect on the expression of IAP members such as XIAP and CIAP-1. As shown in Fig. 4d, bortezomib treatment resulted in down-regulation of XIAP cIAP-1 and survivin. These findings suggest that bortezomib-mediated apoptosis involved these IAP proteins in CML cells.

### Anti-leukemic effects of bortezomib in CML cell lines

**Fig. 5:**
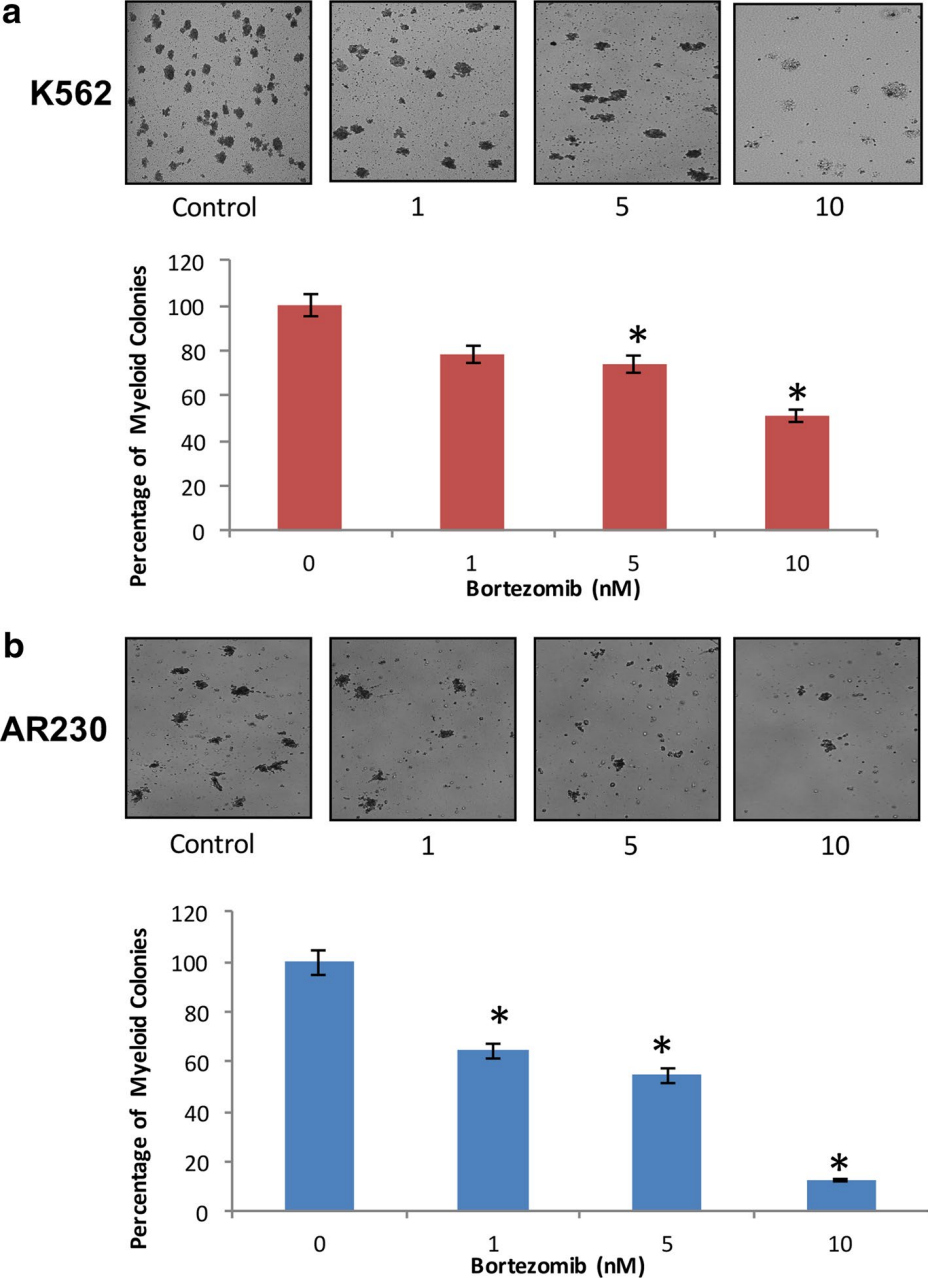
Antileukemic effects of bortezomib on CML cells. **a** K562 and **b** AR230 cells were plated on methyl-cellulose with 1, 5 and 10 nm of bortezomib for 10 days. Cells were counted manually under the microscope. The *bar graphs* represent the percentage of myeloid colonies. The *graph* represents the mean of the two independent experiments *p < 0.05

To assess the anti-leukemic effects induced by bortezomib treatment in a more patho-physiologically relevant approach, we evaluated the effects on leukemic progenitors (CFU-L) using clonogenic assays in methylcellulose. Treatment of K562 and AR230 cells with bortezomib resulted in greater inhibition of CFU-L colony growth of leukemic precursors in both cell lines. The effect was dose-dependent, and a significant decrease was observed at 10 nm treatment with of bortezomib (Fig. 5).

## Discussion

Many of the recent studies have reported the essential role that SKP2 play in the degradation of p27Kip1 in association with various cancers [15, 44]. Degradation of p27Kip1, a mammalian cyclin-dependent kinase inhibitor, is an essential step for the necessary transition from quiescence to the proliferative state. The ubiquitination of p27Kip1 enabled by SKP2 protein leads to its rapid proteasome-mediated degradation [8]. A substantial number of studies account for the interaction between these two proteins whereby it was testified that the expression of SKP2 was inversely linked with p27Kip1 levels [12, 14]. Taking this into account, in the current study, we sought to investigate the relationship between SKP2 and p27Kip1 in the pathogenesis of CML. The protein p27KIP1 is a primary target of SKP2 in context to cell growth and survival. It has been shown that SKP2 knockout mouse embryonic fibroblasts (MEFs) grow significantly slower than wild-type mouse MEFs suggesting an important role of SKP2 in cell proliferation [45]. It is further reported that MEFs devoid of SKP2 have an amplified expression of p27Kip1, which provides the genetic evidence that SKP2 function leads to the degradation of p27Kip1 tumor suppressor protein. Furthermore, in various human cancers the increased expression of SKP2 and reduced levels of p27Kip1 have been linked to poor prognosis [46].

It is worth elucidating that SKP2 down-regulation leading to the p27KIp1 accumulation represents an important underlying mechanism for the antiproliferative effect of the large anticancer compounds, including bortezomib [15, 19, 47] and imatinib [48]. Our findings thus add bortezomib to the growing list of anticancer compounds that target the SKP2–p27KIP1 axis to inhibit the growth and survival of many cancers [14, 47, 49].

Proteasome inhibitors were reported to stabilize many of the cellular proteins, primarily inducing cell cycle arrest followed by programmed cell death. It was observed from the current study that the treatment of CML cells with bortezomib induced a dose-dependent inhibition of cell viability via induction of apoptosis. Bortezomib-mediated reduction of SKP2 led the up-regulation of p27Kip1—a known target of proteasome [15, 19, 47]. Cycloheximide chase experiment strongly suggested that bortezomib stabilizes the expression of p27kip1. Furthermore, silencing of SKP2 expression by siRNA resulted in increased levels of p27Kip1, strongly suggesting a potential role of bortezomib-mediated apoptosis in CML cell lines.

The mechanism of bortezomib-mediated apoptosis in CML cells is still not known. Recent studies have implicated that, the down-regulation of SKP2 by cell adhesion to stroma cells, leads to up-regulation of p27Kip1 [50]. It could be inferred from these studies that regulation of SKP2 happens at both translational and post-translational levels. In our present study, we speculate that bortezomib-induced down-regulation of SKP2 follows a similar mechanism; however, further investigations in this direction is needed. Apoptosis involves a number of anti- and pro-apoptotic signaling proteins in its execution [51]. In the current study inhibition of ubiquitin–proteasomal pathway by bortezomib in CML cells leads to induction of apoptosis via caspase-8 activation followed by truncation of Bid that resulted in a conformational change in the proapoptotic protein, Bax. Bax protein has been shown to affect the integrity of mitochondrial membrane leading to the loss of its potential resulting in the release of cytochrome c into the cytosol. Bortezomib-induced loss of mitochondrial membrane potential helps to achieve the release of cytochrome c from the cristae regions to the cytosol. The cytochrome c release leads to the formation of apoptosome by interaction with apaf1 and caspase-9, which in turn is resulting in sequential activation of caspase-3 and PARP [52]. Furthermore, it was observed that bortezomib treatment in CML cell lines led to reduced expression of inhibitory apoptotic proteins such as XIAP, cIAP1, and survivin implicating the role of IAPs in the activation of caspase-9 and caspase-3 in bortezomib-induced apoptosis. The data suggest that inhibition of ubiquitin–proteasome pathway in CML cells induces apoptosis via caspase cascade activation.

Altogether, the results from our current study establish that SKP2 ubiquitin–proteasome plays a pivotal role in the growth and survival of CML cells. Bortezomib-mediated down-regulation of SKP2 expression leads to the accumulation and stabilization of p27Kip1, thereby inducing apoptosis in CML cells through the release of cytochrome c from the mitochondria and activation of downstream caspase cascades. We anticipate that our present work showing bortezomib mediated downregulation of SKP2 as a result of its correlation with p27Kip1 stabilization and inhibition of CML cell proliferation is a novel finding that can have potential implications for future pre-clinical and clinical studies in CML. The precise mechanism of bortezomib mediated downregulation of SKP2 is not known. Recently SKP2 has been shown to be downregulated by cell adhesion to stroma cells leading upregulation p27kip1 [50]. These studies further suggested that regulation of SKP2 occurs at both translational and posttranslational levels. We speculate that bortezomib-induced downregulation of SKP2 follows similar mechanisms, however it needs further investigations. Indeed, it can open up new avenues of investigations aimed at determining the efficacy of an innovative approach for targeted therapy of the CML subset showing alteration in the ubiquitin–proteasome system with inhibitors of proteasome pathways.

## Conclusions

In conclusion, our results show that bortezomib-mediated inhibition of cell proliferation of CML cell lines is associated with down-regulation of SKP2 with concomitant up-regulation/stabilization of p27Kip1. Furthermore, CML cells treated with bortezomib exhibited inhibition of cell growth and induced apoptosis by activation of the caspase cascade as well as the disruption of the mitochondrial equilibrium. Altogether our findings suggest that bortezomib-mediated anti-proliferative effect of CML cells is novel, which implies that this proteasome inhibitor is of high therapeutic potential that might be used in the strategic intervention of CML.

## Additional files

10.1186/s12967-016-0823-y Bortezomib-mediated induction of early (Annexin +ive and PI −ive cells), late apoptosis (Annexin +ive and PI +ive cells) and necrosis fractions (Annexin −ive and PI +ive cells) in K562 (**A**) and AR230 (**B**) cells. Cells were treated with 10, 25 and 50 nM of bortezomib for 24 h and cells were subsequently stained with flourescein-conjugated annexin-V and propidium iodide (PI) and analyzed by flow cytometry. The graph displays the mean ± SD (standard deviation) of three independent experiments for all the doses. ******* p < 0.001. Bortezomib mediated induction apoptosis in K562 (**C**) and AR230 (**D**) cells. The percentage of apoptotic cells relative to control population is shown. The graph displays the mean ± SD (standard deviation) of three independent experiments for all the doses.
10.1186/s12967-016-0823-y Bortezomib treatment induces double-stranded breaks in K562 cells (**A**). Cells were treated with 10, 25 and 50 nM of bortezomib for 24 h and cells were subsequently stained with H2AX (pS139)-Alexa Fluor 647 antibody as described in “Methods” section and then analyzed by flow cytometry. The graph displays the mean ± SD (standard deviation) of three independent experiments for all the doses. ******* p < 0.001. (B) Shown are images from fluorescence microscope of Bortezomib tretaed K562 cells stained with DAPI. K562 cells were treated with 10, 25 and 50 nm of bortezomib for 24 h and nuclei were subsequently stained with 4’,6-diamidino-2-phenylindole (DAPI) and examined by fluorescence microscopy. Imaging was performed the next day on Zeiss axio imager microscope using 63 × /1.25 oil objectives.
10.1186/s12967-016-0823-y Bortezomib treatment down-regulated the expression of SKP2 and increased the level of p27Kip1. LAMA-84 cells were treated with various doses of bortezomib for 24 h as indicated. After cell lysis, equal amounts of proteins were separated by SDS-PAGE, transfered to PVDF membrane, and immuno-blotted with antibodies against SKP2, p27Kip1 and GAPDH as indicated.
10.1186/s12967-016-0823-y Bortezomib mediated activation of caspase-3 and PARP cascade in LAMA-84 cells. AR230 and K562 cells were treated with and without 10, 25 and 50 nm of bortezomib for 24 h. Cells were lysed and 25 μg of proteins were separated on SDS-PAGE, transferred to PVDF membrane, and immunoblotted with antibodies against caspase-3, PARP and GAPDH.
