# Supplementary figures and images for: Bortezomib-mediated downregulation of S-phase kinase protein-2 (SKP2) causes apoptotic cell death in chronic myelogenous leukemia cells
Source: J Transl Med. 2016 Mar 9;14:69. doi: 10.1186/s12967-016-0823-y (PMC4784454; doi:10.1186/s12967-016-0823-y)

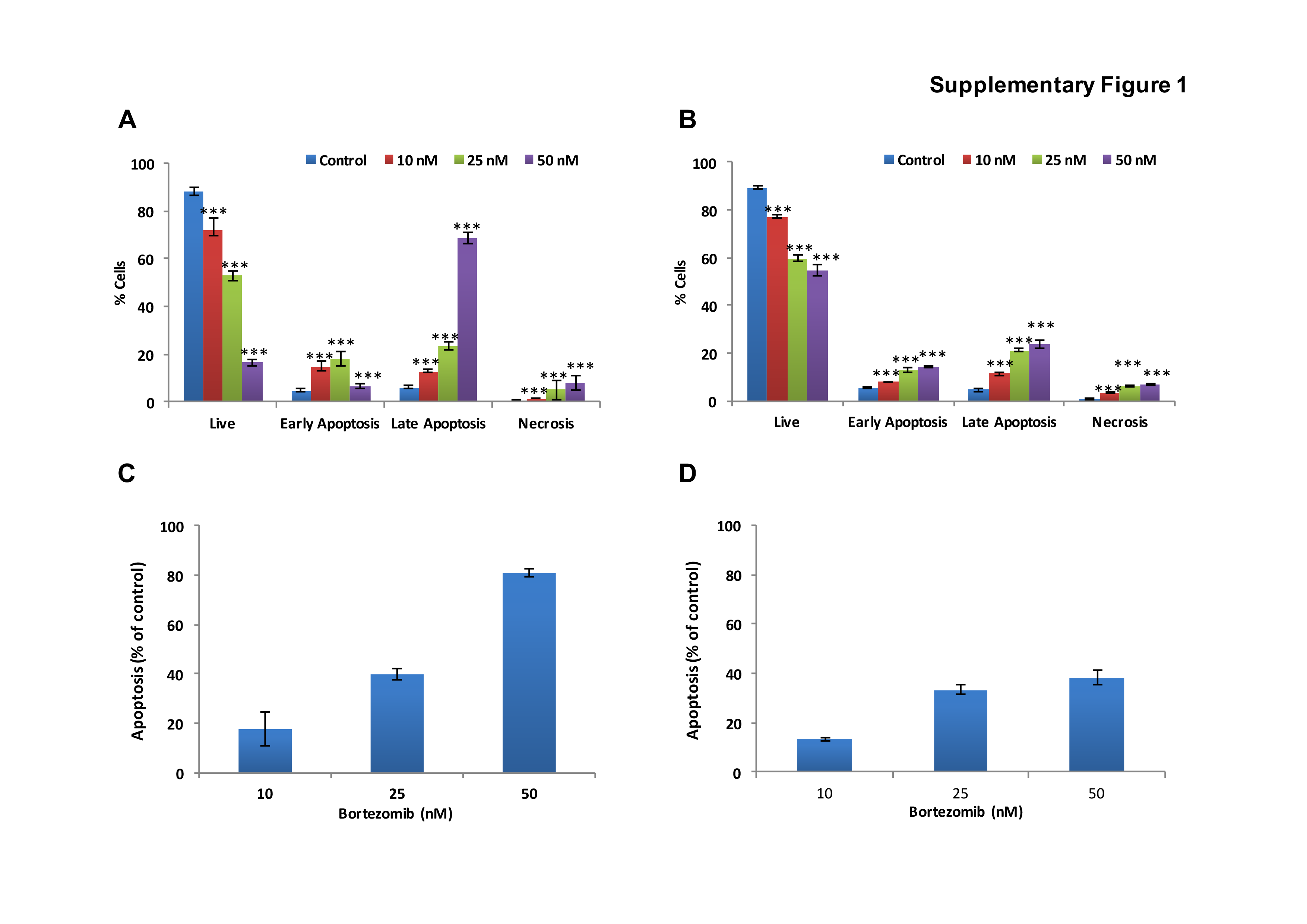

Supplement: Supplementary file 1 — 10.1186/s12967-016-0823-y Bortezomib-mediated induction of early (Annexin +ive and PI −ive cells), late apoptosis (Annexin +ive and PI +ive cells) and necrosis fractions (Annexin −ive and PI +ive cells) in K562 (A) and AR230 (B) cells. Cells were treated with 10, 25 and 50 nM of bortezomib for 24 h and cells were subsequently stained with flourescein-conjugated annexin-V and propidium iodide (PI) and analyzed by flow cytometry. The graph displays the mean ± SD (standard deviation) of three independent experiments for all the doses. *** p < 0.001. Bortezomib mediated induction apoptosis in K562 (C) and AR230 (D) cells. The percentage of apoptotic cells relative to control population is shown. The graph displays the mean ± SD (standard deviation) of three independent experiments for all the doses. [file 12967_2016_823_MOESM1_ESM.tif]

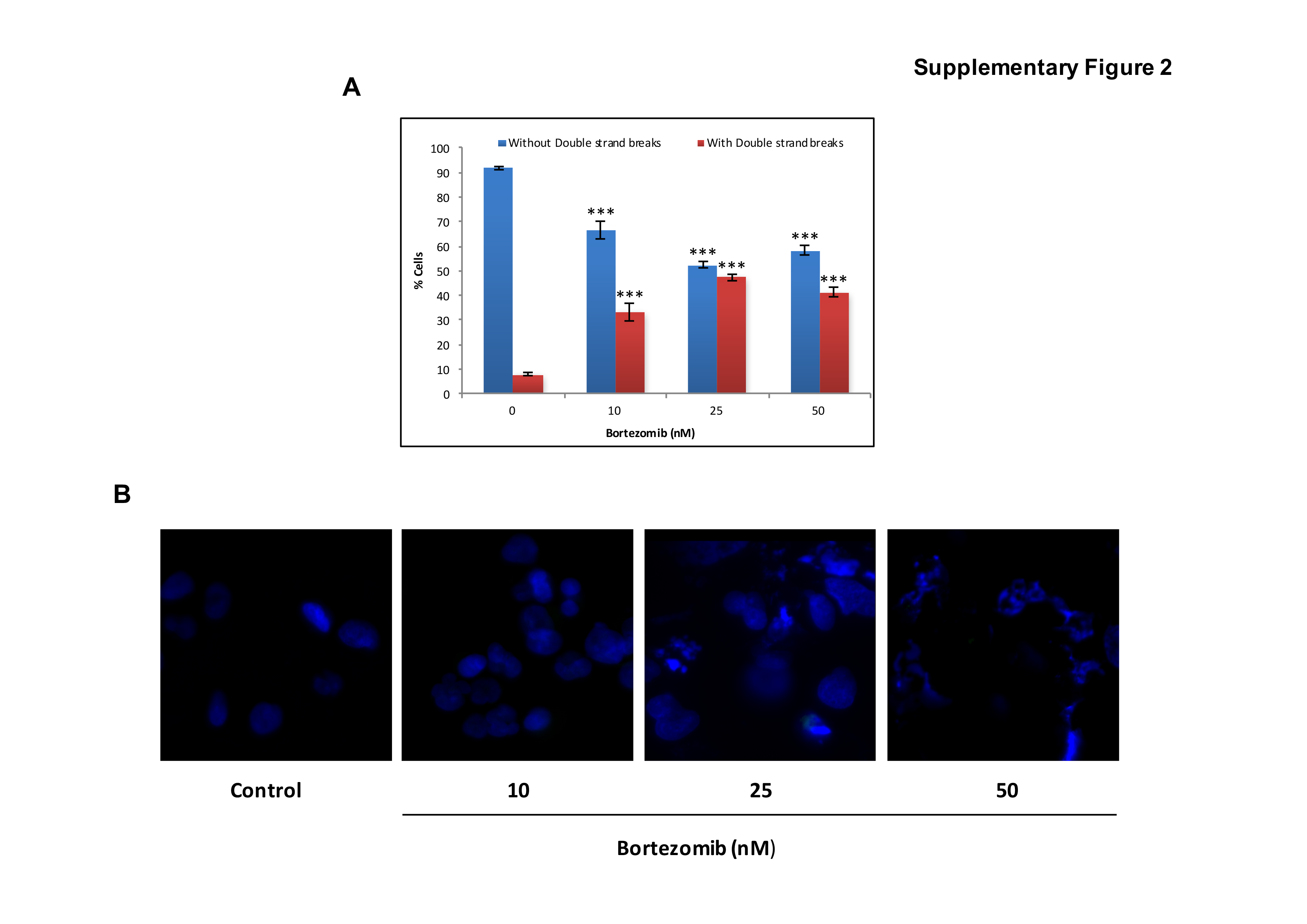

Supplement: Supplementary file 2 — 10.1186/s12967-016-0823-y Bortezomib treatment induces double-stranded breaks in K562 cells (A). Cells were treated with 10, 25 and 50 nM of bortezomib for 24 h and cells were subsequently stained with H2AX (pS139)-Alexa Fluor 647 antibody as described in “Methods” section and then analyzed by flow cytometry. The graph displays the mean ± SD (standard deviation) of three independent experiments for all the doses. *** p < 0.001. (B) Shown are images from fluorescence microscope of Bortezomib tretaed K562 cells stained with DAPI. K562 cells were treated with 10, 25 and 50 nm of bortezomib for 24 h and nuclei were subsequently stained with 4’,6-diamidino-2-phenylindole (DAPI) and examined by fluorescence microscopy. Imaging was performed the next day on Zeiss axio imager microscope using 63 × /1.25 oil objectives. [file 12967_2016_823_MOESM2_ESM.tif]

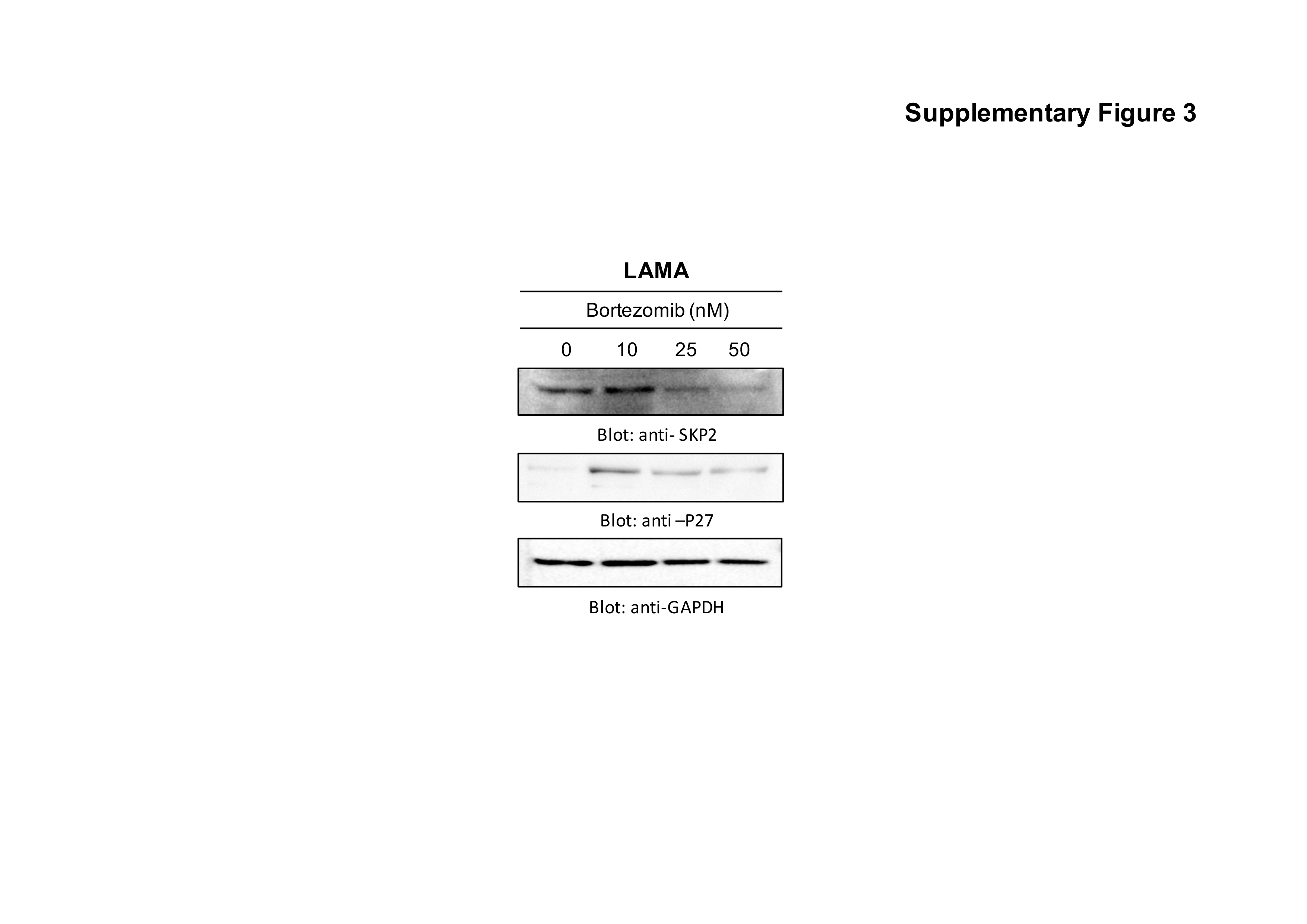

Supplement: Supplementary file 3 — 10.1186/s12967-016-0823-y Bortezomib treatment down-regulated the expression of SKP2 and increased the level of p27Kip1. LAMA-84 cells were treated with various doses of bortezomib for 24 h as indicated. After cell lysis, equal amounts of proteins were separated by SDS-PAGE, transfered to PVDF membrane, and immuno-blotted with antibodies against SKP2, p27Kip1 and GAPDH as indicated. [file 12967_2016_823_MOESM3_ESM.tif]

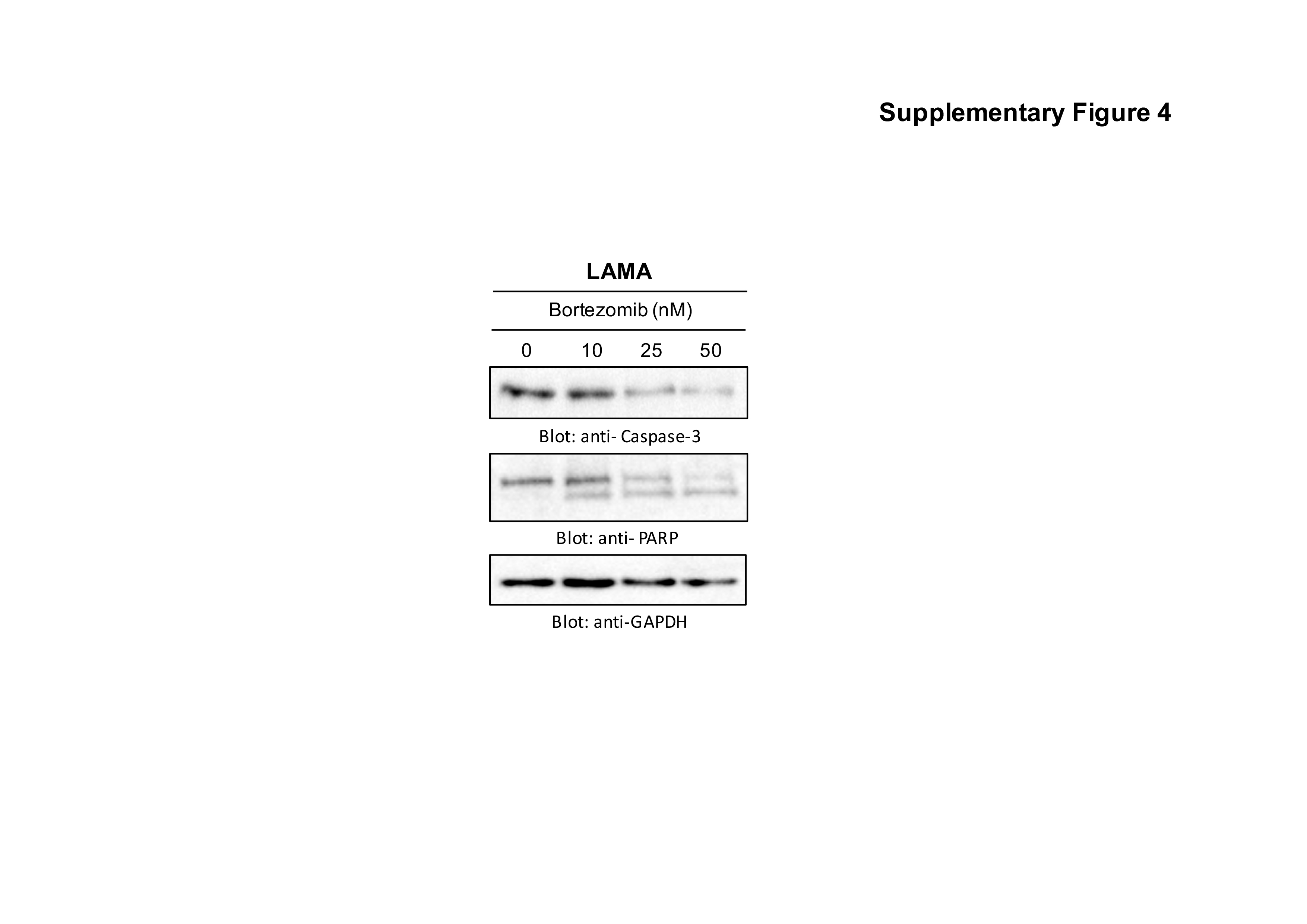

Supplement: Supplementary file 4 — 10.1186/s12967-016-0823-y Bortezomib mediated activation of caspase-3 and PARP cascade in LAMA-84 cells. AR230 and K562 cells were treated with and without 10, 25 and 50 nm of bortezomib for 24 h. Cells were lysed and 25 μg of proteins were separated on SDS-PAGE, transferred to PVDF membrane, and immunoblotted with antibodies against caspase-3, PARP and GAPDH. [file 12967_2016_823_MOESM4_ESM.tif]
